# Enhanced sub-1 eV detection in organic photodetectors through tuning polymer energetics and microstructure

**DOI:** 10.1126/sciadv.adh2694

**Published:** 2023-06-07

**Authors:** Polina Jacoutot, Alberto D. Scaccabarozzi, Davide Nodari, Julianna Panidi, Zhuoran Qiao, Andriana Schiza, Alkmini D. Nega, Antonia Dimitrakopoulou-Strauss, Vasilis G. Gregoriou, Martin Heeney, Christos L. Chochos, Artem A. Bakulin, Nicola Gasparini

**Affiliations:** ^1^ Department of Chemistry and Centre for Processable Electronics, Imperial College London, London W12 0BZ, UK.; ^2^ Center for Nano Science and Technology@PoliMi, Istituto Italiano di Tecnologia, via Raffaele Rubattino 81, Milano 20134, Italy.; ^3^ Institute of Chemical Biology, National Hellenic Research Foundation, 48 Vassileos Constantinou Avenue, Athens 11635, Greece.; ^4^ Clinical Cooperation Unit Nuclear Medicine, German Cancer Research Center, 69120 Heidelberg, Germany.; ^5^ Advent Technologies SA, Stadiou Street, Platani, Rio, Patras 26504, Greece.; ^6^ King Abdullah University of Science and Technology (KAUST), KAUST Solar Center (KSC), Thuwal 23955, Saudi Arabia.

## Abstract

One of the key challenges facing organic photodiodes (OPDs) is increasing the detection into the infrared region. Organic semiconductor polymers provide a platform for tuning the bandgap and optoelectronic response to go beyond the traditional 1000-nanometer benchmark. In this work, we present a near-infrared (NIR) polymer with absorption up to 1500 nanometers. The polymer-based OPD delivers a high specific detectivity D^*^ of 1.03 × 10^10^ Jones (−2 volts) at 1200 nanometers and a dark current *J*_d_ of just 2.3 × 10^−6^ ampere per square centimeter at −2 volts. We demonstrate a strong improvement of all OPD metrics in the NIR region compared to previously reported NIR OPD due to the enhanced crystallinity and optimized energy alignment, which leads to reduced charge recombination. The high D^*^ value in the 1100-to-1300-nanometer region is particularly promising for biosensing applications. We demonstrate the OPD as a pulse oximeter under NIR illumination, delivering heart rate and blood oxygen saturation readings in real time without signal amplification.

## Teaser

SWIR polymer-based organic photodetectors with high detectivity and fast response speed for biometric applications are developed.

## INTRODUCTION

Light detection is at the core of modern technology. With the growing variety of electronic devices to support the Internet of Things services, the research community needs to address the rising demand. By 2030, more than 25 billion devices are forecasted to be connected, and about 15% of the global value is allocated to the health sector (*1*, *2*). Visible and infrared (IR) photodetectors based on silicon and indium gallium arsenide (InGaAs) dominate the current market thanks to their outstanding optoelectronic properties, with specific detectivity exceeding 10^12^ Jones. Notwithstanding, their lack of flexibility and costly manufacturing processes have left room for other emerging technologies such as organic photodetectors (OPDs) (*3*, *4*). Organic optoelectronics can address the fast-growing demand for portable, lightweight, cost-effective sensors that are flexible and easy to integrate and scale (*5*–*8*). Among notable benefits of organic semiconductors are broad and tunable absorption, solution processing, and a choice of deposition techniques onto soft, curved, or large surface areas. Moreover, these advantages over the established photodetector technology make OPD technology a desirable choice for large-area flexible imagers (*9*, *10*), on-the-go monitoring (*11*), and skin-grafted sensors and bioelectronics (*12*).

To overcome the exciton binding energy in organic semiconductors, a bulk heterojunction device architecture is realized by blending electron-donating (D) and electron-accepting (A) organic molecules in the photoactive layer (*13*, *14*). Now, the widely accepted benchmarks for IR OPDs consist of conjugated donor polymers and small-molecule non-fullerene acceptors (*15*, *16*). In the visible region, the photoactive layer materials arise from broad and intense research on organic photovoltaic technology, which helps visible OPDs to achieve metrics comparable with benchmark inorganic photodetectors (*9*, *17*, *18*). However, we rarely see IR materials with absorption onset above 1000 nm, as the solar spectrum irradiance drops off strongly in the IR. The additional difficulty in going further into the IR region arises from synthetic challenges in developing solution-processable, scalable IR–absorbing organic materials (*19*–*21*).

D-A polymers with absorption extending into the IR carry a vast potential for the OPD community (*22*–*24*). These near-infrared (NIR) polymers provide a wide scope for synthetic chemists to fine-tune the energetic levels, microstructure, solubility, and other properties (*23*, *25*). The electron-deficient A moiety largely determines the lowest unoccupied molecular orbital (LUMO) of the polymer, while the electron-rich D unit strongly influences the highest occupied molecular orbital (HOMO) energy. The former, for instance, can be used to lower the LUMO through the stabilization of the quinoid form of the acceptor unit (*23*, *26*). In our previous work, we demonstrated an NIR OPD based on thiadiazoloquinoxaline-thiophene (TQ-T) polymer and IEICO-4F with photocurrent response up to 1800 nm (*27*). However, those photodetectors suffered from high *J*_d_ under reverse bias. Given the ultranarrow optical bandgap of TQ-T polymer, it is expected that nonradiative recombination strongly limits the specific detectivity (D*) of the device, while the energetic alignment with the charge blocking layers results in high injection current at the electrodes at reverse bias (*28*, *29*). The resulting D* is far below the established background-limited IR photodetection detectivity limits for OPDs (*30*).

For biosensing and imaging applications, it is advantageous to focus on improving D* in the second NIR detection window (1100 to 1300 nm), where tissue penetration is greater than with visible light, and light attenuation is minimized (*31*–*33*). To suppress the carrier injection from the electrodes at reverse bias, a deeper HOMO level of D is beneficial for increasing the injection barrier and forming a better ohmic contact with hole-transporting layers (HTLs), i.e., poly(3,4-ethylenedioxythiophene) polystyrene sulfonate (PEDOT:PSS) or molybdenum oxide (MoO*_x_*) (*34*, *35*). Therefore, a strategy for OPD optimization should include fine-tuning of D molecular orbital energy levels and improving carrier mobility through control of the microstructure and optimized blend morphology (*36*–*38*).

In this work, we report a newly synthesized IR polymer that, in combination with IEICO-4F, realized an OPD with high D* of 10^10^ Jones at 1200 nm. We compare the OPD and material parameters with those previously reported for the TQ-T:IEICO-4F system and demonstrate a strong performance improvement. We assign this improvement to higher crystallinity of the newly synthesized polymer, which affords improved molecular packing and carrier mobility both in the pristine material and in the blend, while its deeper HOMO minimizes the effect of dark charge injection at reverse bias. Through a careful analysis of the charge generation dynamics in the blend and of the OPD response kinetics, we observe efficient charge generation under NIR illumination within <2 ps, accompanied by rapid 2- to 5-μs rise and fall times of the photoresponse owing to the higher carrier mobility and suppressed recombination in the blend. The resulting specific detectivity is among the highest reported values for solution processable OPDs in the second NIR detection window (*17*, *22*, *24*, *39*). This allows us to demonstrate an application of the sensor as a pulse oximeter, which successfully and accurately measures heart rate and blood oxygen saturation under NIR illumination.

## RESULTS

### Materials characterizations

**Fig. 1.**
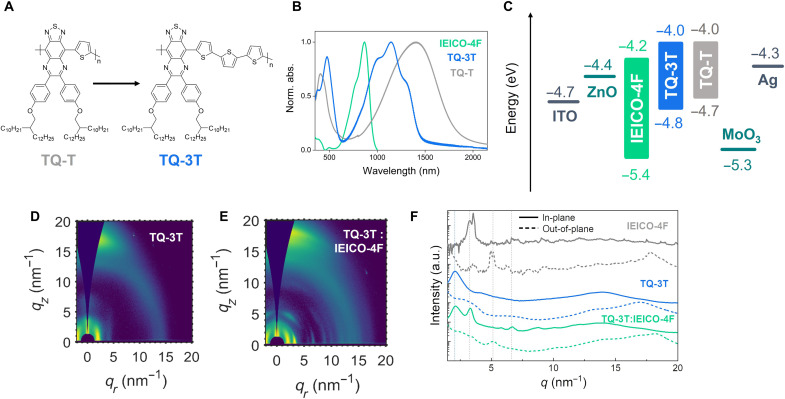
TQ-3T polymer structure and OPD materials characterization. (**A**) Chemical structure of low bandgap conjugated polymers TQ-T and TQ-3T. (**B**) Normalized visible-IR absorption spectra of conjugated donor polymers and non-fullerene acceptor IEICO-4F. (**C**) Energy diagram of the materials used in OPD measured by air photoelectron spectroscopy (APS), with workfunction values of 4.7 and 4.6 eV for TQ-3T and TQ-T, respectively, obtained by Kelvin probe measurements. 2D GIWAXS maps of (**D**) TQ-3T and (**E**) TQ-3T:IEICO-4F; (**F**) in-plane and out-of-plane profiles of pristine films and the D:A blend. a.u., arbitrary units.

Figure 1A depicts the chemical structures of two solution-processable conjugated polymers based on the TQ building block. We previously demonstrated an OPD based on TQ-T push-pull polymer blended with IEICO-4F to achieve spectral responsivity up to 1800 nm (*27*). We showed that the effect of blend morphology played a key role in achieving higher D* in the short-wave IR region beyond 1000 nm. However, those OPDs suffered from relatively high dark currents *J*_d_ due to the energetic alignment and the amorphous nature of the ultralow bandgap polymer. Here, we report a newly synthesized solution-processable IR polymer TQ-3T with three electron-rich thiophene units extending along the backbone. The new polymer was synthesized by Stille cross-coupling polymerization reaction (outlined in figs. S1 to S6).

Normalized absorption spectra of TQ-3T, TQ-T, and IEICO-4F are displayed in Fig. 1B. Peak absorption for TQ-3T is observed at 1150 nm, and the absorption onset occurs at 1470 nm, corresponding to an optical bandgap of 0.84 eV. The formation of multiple absorption peaks in TQ-3T suggests a higher degree of crystallinity, which will be discussed later in the morphology section. Relative to TQ-T, in the new polymer, we observe a deepening of the HOMO energy level from −4.6 to −4.7 eV, according to photoelectron spectroscopy measurements in air (fig. S7). At a first glance, it is expected that the introduction of an additional (electron-rich) T comonomers leads to a slight deepening of the HOMO. However, we speculate that this is due to the unusual properties of the TQ monomer. TQ is known to promote quinoidal character along the backbone, assisted by the formal aromatization of the pyrazine ring, leading in some cases to an open-shell ground state character and very low bandgaps (*40*, *41*). By extending the number of aromatic comonomers from one (T) to three (3T), we believe that the degree of quinoidal character of the polymer is reduced, leading to the measured changes in energy levels. This is beneficial in reducing the leakage current from carrier injection at reverse bias (*9*, *42*). By having better energy alignment with the MoO*_x_* HTL, a better Ohmic contact is formed in the inverted OPD devices than previously with TQ-T polymer. While the introduction of the two more electron-rich T donor units deepened the HOMO energy in TQ-3T, the LUMO energy calculated as HOMO + *E_g_* remains similar in the two polymers with an identical TQ acceptor unit.

Another effect of the backbone modification is the improved crystal order of the TQ-3T thin films, which was observed with grazing-incidence wide-angle x-ray scattering (GIWAXS) measurements. Figure 1 (D to F) illustrates the two-dimensional (2D) GIWAXS patterns for the D:A blend and the pristine materials. We have previously shown that TQ-T has a semicrystalline structure with a low degree of order, which also disrupts the high packing order of IEICO-4F in the D:A blend (*27*). Although a peak at 3.1 nm^−1^ associated with IEICO-4F was also observed in the TQ-T:IEICO-4F blend, this blend remains largely amorphous. Compared to its predecessor, TQ-3T displays an increased order and anisotropy, with a preferential face-on orientation in a thin film. The high crystal order of IEICO-4F and TQ-3T is also maintained in the blend. Sharp in-plane diffraction peaks at 2 and 3 nm^−1^ are ascribed to TQ-3T and IEICO-4F, respectively. The out-of-plane peak for IEICO-4F at 5 nm^−1^ is preserved in the blend, along with other diffractions from the polymer at lower angles. Therefore, TQ-3T:IEICO-4F blend shows the same crystal quality and orientation of its neat components.

To get an insight into the materials’ charge transport properties, organic thin-film transistors (OTFTs) were fabricated in bottom-contact, top-gate architecture. Both TQ-T and TQ-3T films showed high ambipolarity (fig. S8), with the former exhibiting mainly n-type and the latter p-type character. TQ-3T achieved a substantially higher hole mobility of 7 × 10^−2^ cm^2^ V^−1^ s^−1^ in the saturation regime when compared with TQ-T, which reached 3.5 × 10^−3^ cm^2^ V^−1^ s^−1^. Regarding their n-type operation, TQ-T showed highest electron charge transport in the order of 3 × 10^−2^ cm^2^ V^−1^ s^−1^, whereas TQ-3T reached a mobility of 3 × 10^−3^ cm^2^ V^−1^ s^−1^. The mostly p-type operation of TQ-3T devices can be assigned to the deeper HOMO and workfunction value (see Fig. 1C), which reduces the energetic barrier with the source/drain electrodes. Moreover, the relatively high hole mobility of TQ-3T can also be assigned to the improved microstructure, as observed from the GIWAXS measurements (*43*, *44*).

### Near-infrared OPDs

**Fig. 2.**
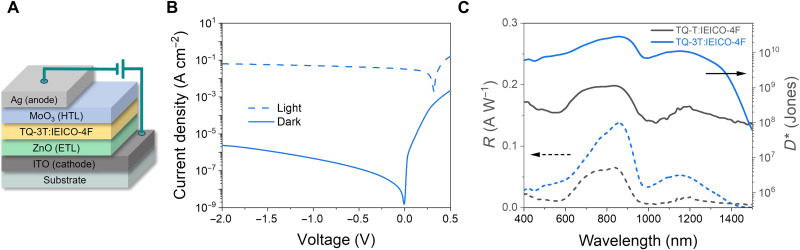
OPD architecture and performance. (**A**) Inverted OPD device structure of the TQ-3T–based device. (**B**) Current-voltage characteristics of TQ-3T:IEICO-4F OPDs under light and dark conditions. (**C**) Responsivity and specific detectivity at −2 V applied bias.

To evaluate the ability of TQ-3T to convert NIR light into current, we fabricated OPDs with an inverted device architecture by blending the IR polymer with IEICO-4F in a 1:1 optimized ratio (Fig. 2A). Photodetectors require efficient light-to-current conversion and low *J*_d_ for good light detection. One of the key methodologies for improving the photodetector’s sensitivity to light, i.e., increasing signal-to-noise ratio, linear dynamic range (LDR), and specific detectivity, is the minimization of the leakage current generated at reverse bias. A well-established practice is to use HTL and electron-transporting layer to suppress parasitic currents across the device under reverse bias, which we also adopted in this work. Effective suppression of *J*_d_ can also be achieved by deepening the HOMO of D or shallowing of A LUMO levels, which, unfortunately, presents a trade-off for ultralow bandgap materials (*42*, *45*–*47*).

Previously studied system TQ-T:IEICO-4F suffered from the relatively high dark currents of 8.4 × 10^−5^ A cm^−2^ (fig. S9), which were improved in this work by suppressing dark current injection from the HTL. As a result, in Fig. 2B, we observe dark current of 2.3 × 10^−6^ A cm^−2^ at −2 V with TQ-3T:IEICO-4F OPD. Under the illumination conditions, we observe higher photocurrent (*J*_L_) values of 6.3 × 10^−2^ A cm^−2^ at −2 V, and an open circuit voltage of 0.32 V under one sun equivalent illumination. The improved OPD sensitivity in TQ-3T–based devices compared to TQ-T is demonstrated by the spectral responsivity (*R*) in Fig. 2C.

The OPD’s photocurrent response to incident photons of varying energy is known as responsivity *R*. We calculated *R* from the following equation

$$R=\frac{J_{\mathrm{ph}}}{P_{in}}=\eta \frac{\lambda q}{hc}$$ (1)

where *P_in_* is the power, λ is the wavelength of incident light, η is the external quantum efficiency, *q* is the elementary charge, *h* is the Planck’s constant, and *c* is the speed of light.

The spectral responsivity covers the NIR region up to 1500 nm, with *R* of 0.05 A W^−1^ measured at 1200 nm. This is significantly higher across the whole NIR spectral range than previously observed with TQ-T:IEICO-4F.

A key parameter for light-sensing applications is the specific detectivity, D*, calculated according to the equation below

$$D^{*}=\frac{R\sqrt{A\text{Δ}f}}{i_{\mathrm{noise}}}$$ (2)

where *A* is the photoactive area of the device, Δ*f* is the detection bandwidth, and *i*_noise_ is the noise current. To avoid an overestimation of D*, the total experimental noise currents should be considered in the calculation (figs. S10 and S11). Because other sources of white noise, such as flicker and thermal noise, provide a significant contribution to the overall noise of the device, *J*_d_ cannot be considered as its main contributor (*48*). We calculated D* to be 1.03 × 10^10^ Jones at 1200 nm under −2-V applied bias.

So far, we have presented steady-state characteristics of the OPD. However, to compete with existing benchmarks for imaging, video, or communication applications, we need to meet the minimum detection speed requirements of 10 kHz (*49*). Therefore, we examine dynamic characteristics by measuring the electrical bandwidth and rise *t*_r_ and fall *t*_f_ times of the OPD with IR light illumination. The bandwidth of a photodetector is known as a frequency of an incident modulated light at which the photocurrent response of the device has diminished by 3 dB from its low-frequency value. It is limited by the transit time of carriers to the electrodes and parasitic capacitance in the photoactive layer.

$$\mathrm{Damping}\;(\mathrm{dB})=20\log\frac{i(f_{3\mathrm{dB}})}{i_{\max}}$$ (3)

**Fig. 3.**
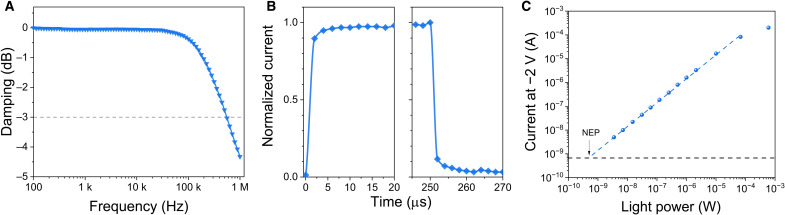
OPD response speed and dynamic range. Dynamic measurements with TQ-3T:IEICO-4F all performed at 940-nm illumination and −2 V applied bias. (**A**) Cut-off frequency at −3 dB. (**B**) Transient photocurrent measurements with rise and fall times. (**C**) LDR measurements and extrapolation of the LDR into the noise floor, with calculation of the NEP.

Dynamic measurements were performed to evaluate the speed of the OPD. Figure 3A illustrates the electrical bandwidth of 470 kHz under 940-nm light at −2 V. Similar values were obtained under 1100- and 1300-nm illumination (fig. S12), confirming the high response speed of these NIR devices. The OPD demonstrates 2 μs *t*_r_ and *t*_f_ under 940-nm illumination and 3 and 5 μs *t*_r_ and *t*_f_, respectively, under 1100-nm light (fig. S13). At applied reverse bias, an OPD is operated in the linear light intensity regime, known as LDR. LDR has been calculated to be 84 dB at 940 nm, which, again, is an improvement over the TQ-T–based system (Table 1). Last, the extrapolation of the LDR into the noise floor allowed the calculation of the noise equivalent power (NEP). We obtained a NEP value of 0.3 nW/√Hz, which results in D* values of 7 × 10^9^ Jones at 940 nm. This value is in line with the D* calculated in Fig. 2C.

**Table 1.** Comparison of the OPD figures of merit for TQ-3T– and TQ-T–based devices.

Active layer	*E*_*g*_ (eV)	λ_onset_ (nm)	*J*_d_ (A cm^−2^)	D* (Jones)	*R* (A W^−1^)	Cut-off frequency (kHz)	LDR (dB)
TQ-3T:IEICO-4F	0.84	1470	2.3 × 10^−6^	1.03 × 10^10^ at 1200 nm	0.05 at 1200 nm	470 kHz	84 at 940 nm
TQ-T:IEICO-4F	0.67	1800	8.4 × 10^−5^	3.04 × 10^8^ at 1200 nm	0.02 at 1200 nm	100 kHz	46 at 940 nm

From Table 1, we can clearly see an improvement across most OPD parameters, with the only exception of responsivity extending further in the IR window in the TQ-T–based system. We attribute the improved OPD performance of the TQ-3T blend to an enhanced crystalline order and better energy alignment in the device to lower the dark current contributions, resulting in higher detectivity across the entire spectral window.

### Ultrafast spectroscopy analysis

The photocurrent generation efficiency in the TQ-3T:IEICO-4F can be expected given the low offset of LUMO levels. To elucidate the underlying carrier dynamics in the D:A blend on the ultrafast time scale and kinetic limitations of device performance, we studied the blend and pristine films using pump-probe spectroscopy. Global analysis (GA) was applied to the transient absorption data of the blends to deconvolute varying spectral components.

**Fig. 4.**
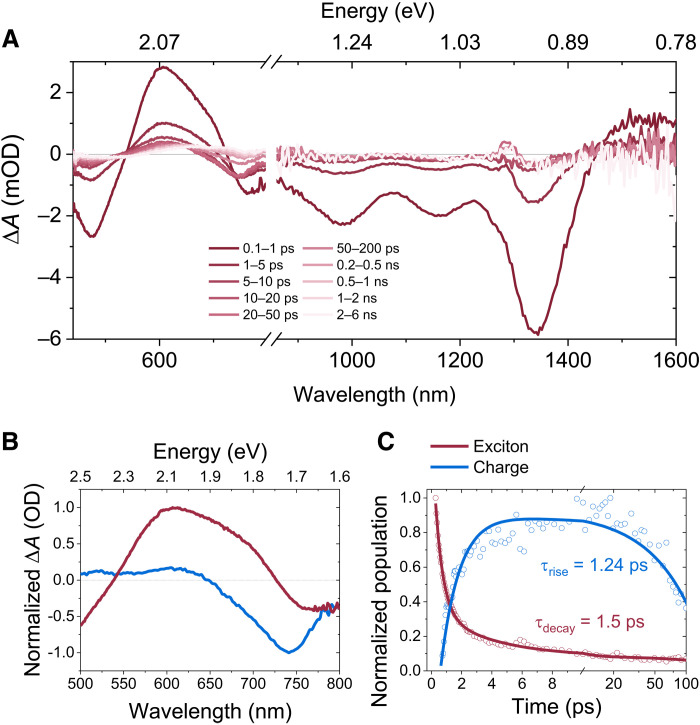
TAS analysis of the OPD blend. (**A**) Broadband visible-IR transient absorption spectra of TQ-3T:IEICO-4F blend upon 1300-nm pump excitation. Decomposed spectra (**B**) and kinetics (**C**) of the two-component GA depicting decaying excitonic and growing charge dynamic. mOD, mean optical density; OD, optical density.

Figure 4A presents a broad (visible-NIR) spectrum of the D:A blend under low-intensity excitation with 1300-nm pump illumination. The IR part of the spectrum is dominated by the ground state bleaching (GSB), representing the ground state absorption of the donor. The ground state absorption of the acceptor is expected in the 700-to-900-nm region. This GSB feature of the A is not observed at early times, as the polymer is selectively excited, but appears at later times when electron is transferred to the A. In addition, the dominant GSB feature from the polymer may be masking the photoinduced absorption from IEICO-4F, which peaks at 1160 nm (fig. S15). The visible part of the probe spectrum presents convoluted spectra from both D and A components as we see a spectral shape distortion and peak shifting beyond ~5 ps. This visible region was closely examined, and a GA was performed using the reference spectra of the pristine materials in figs. S14 and S15. The resulting deconvoluted spectra of the two components are shown in Fig. 4B with their associated kinetics in Fig. 4C. GA is based on a genetic algorithm, which identifies spectral footprints of multiple components within the system, provided that they have different dynamics (*50*). Hence, we were able to identify spectral components associated with the TQ-3T exciton and charges (electrons). A rapid TQ-3T exciton decay within 2 ps is observed from fitting a single exponent. This correlates well with the exponential rise in the second component, which we ascribe to fast charge formation. Through TAS data analysis, we demonstrate that this D:A blend shows rapid exciton separation and charge generation within 2 ps or less. To improve the light-to-current conversion in the NIR region even further, future work will be focusing on the selection of HTLs with a better ohmic contact with the HOMO of the ultralow *E_g_* polymers.

### Real-time heartbeat monitoring and SpO_2_ measurements

Last, we demonstrate a practical application of the new OPD as an NIR pulse oximeter with high-accuracy heart rate monitoring and SpO_2_ saturation. A photoplethysmogram (PPG) was obtained from a healthy young volunteer in a resting state at three different wavelengths: 880, 940, and 1100 nm. PPGs were collected in transmission mode, whereby the fingertip was placed between the light-emitting diode (LED) and the OPD according to the setup described previously (fig. S16). Transmission mode pulse oximetry is useful at locations such as fingers, ears, and toes. For larger body parts, reflectance mode oximeters are a more suitable choice (*51*, *52*).

**Fig. 5.**
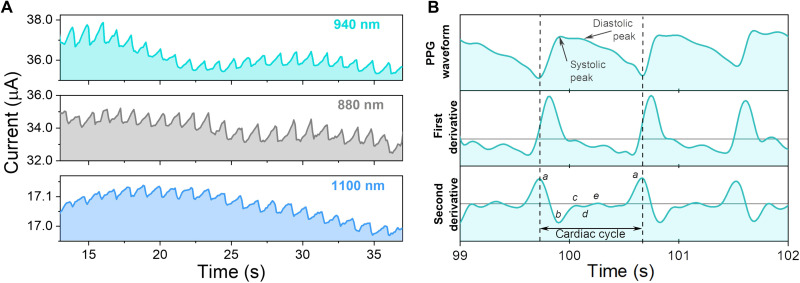
Finger PPG testing under NIR illumination. (**A**) Transmittance PPG collected from a volunteer’s finger under different NIR illumination wavelengths. (**B**) Comparison of PPG waveform and its derivatives, including the second derivative APG, highlighting the key areas of interest in a cardiac cycle.

The LED light was attenuated by pulsating blood in the microvascular network and the surrounding tissue. The transmitted light was directly read out as OPD photocurrent. The resulting waveforms under three different illumination wavelengths are presented in Fig. 5A. Examining the amplitude of the signal and the overall current, the best signal-to-noise ratio was observed under 940-nm light. This is in line with high responsivity at this wavelength.

Despite lower current amplitude at 1100 nm, sharp and well-resolved peaks are still observed, which is valuable in combination with the enhanced tissue penetration and reduced attenuation at this wavelength (*32*, *53*, *54*). From all three waveforms, we calculated average resting heart rates of 58, 56, and 54 bpm, which are in good agreement and were not collected simultaneously. Furthermore, we obtained second derivative waveforms (Fig. 5B), known as the acceleration plethysmogram (APG), which are useful for examining the heart rate variability bypassing contour analysis errors in raw PPG signals (*55*, *56*). APG is also used to extract valuable physiological information about arterial health. The APG waveform has four systolic waves [*a* to *d* in Fig. 5B] and one diastolic wave (*e*). The points indicated in Fig. 5B on the APG wave are known as early systolic positive wave (*a*), early systolic negative wave (*b*), late systolic reincresing wave (*c*), late systolic redecreasing wave (*d*), and early diastolic positive wave (*e*) (*56*, *57*). Peak detection from *a* to *a* can be used to accurately calculate the heart rate, while *b*/*a* ratio is an indicator of arterial stiffness, which increases with age (*58*, *59*). We calculated the average *b*/*a* index for our volunteer to be 0.81, which is in perfect agreement with the *b*/*a* index for the fourth-decade age group in healthy males (*58*, *60*).

PPG is also used in pulse oximetry to extract arterial oxygen saturation (SpO_2_). The pulsatile component AC is superimposed onto the lower-frequency DC component, which are analyzed to obtain the AC/DC ratio at different wavelengths (*61*). The SpO_2_ of 98% was calculated from PPG waveform. The calculations were carried out according to the equation below (*62*)

$$\mathrm{Sp}O_{2}=\frac{\varepsilon_{\lambda_{1},\mathrm{Hb}}-\varepsilon_{\lambda_{2},\mathrm{Hb}}R}{(\varepsilon_{\lambda_{1},\mathrm{Hb}}-\varepsilon_{\lambda_{1},\mathrm{Hb}O_{2}})+(\varepsilon_{\lambda_{2},\mathrm{Hb}O_{2}}-\varepsilon_{\lambda_{2},\mathrm{Hb}})R}$$ (4)

where εHbO_2_ and εHb are absorption coefficients for oxyhemoglobin and deoxyhemoglobin, taken from (*52*) and *R* is the ratio of ac to dc at two different wavelengths. This SpO_2_ value is within range for a healthy young volunteer and demonstrates the new OPD system as an effective pulse oximeter under NIR illumination.

## DISCUSSION

We have synthesized a new IR push-pull polymer based on the TQ-3T backbone with absorption onset at 1470 nm. Using this polymer as a donor material in the bulk heterojunction (BHJ) blend with IEICO-4F, we have realized an NIR OPD with high specific detectivity of 10^10^ Jones at 1200 nm, exceeding previously reported OPD with TQ-T donor polymer by two orders of magnitude. GIWAXS measurements related this improvement to the superior crystalline order of TQ-3T compared to TQ-T polymer. The order was also preserved in the D:A blend where TQ-3T was shown to not disrupt the ordering of IEICO-4F significantly. The improved microstructure in TQ-3T contributed to a reduced charge recombination leading to a faster charge extraction. In addition, efficient photon to charge conversion and fast response speed were observed at different NIR wavelengths, which we attribute to the fast charge generation and extraction in this blend. The novel OPD system demonstrated a reduction in *J*_d_ at reverse bias by an order or magnitude, which we accredited to a better ohmic contact with the HTL through deepening of HOMO (D). Besides an improvement across all the device metrics, we also demonstrated an application as a pulse oximeter, performing heart rate variability and SpO_2_ analysis.

## MATERIALS AND METHODS

### Materials

TQ-3T polymer was synthesized according to the details outlined in the Supplementary Materials. The NFA IEICO-4F was purchased from 1-Material.

### Device fabrication

OPDs were fabricated in an inverted architecture of indium tin oxide (ITO)/ZnO (30 nm)/active layer (120 nm)/MoO*_x_* (10 nm)/Ag (100 nm). Glass substrates prepatterned with ITO were cleaned by sequential sonication in acetone, deionized water, Decon 90 detergent, and propan-2-ol each for 10 min. Following this, an 8-min oxygen plasma treatment was performed. Zinc oxide (ZnO) precursor solution was prepared from zinc acetate dihydrate (219.5 mg), ethanolamine (60 μl), and 2-methoxyethanol (2 ml). This ZnO precursor solution was filtered through a 0.45-μm Acrodisc filter, spin-coated onto the plasma-treated substrates at 4000 rpm/40 s, and annealed at 150°C/20 min. The TQ-3T:IEICO-4F (1:1) and TQ-T:IEICO-4F (1:1) were dissolved in chlorobenzene solutions with total concentrations of 15 and 20 mg/ml, respectively, and stirred overnight at 60°C in a glove box. The active layers were deposited by spin coating at 2000 rpm/40 s in inert conditions and then annealed at 100°C/10 min in the glove box. MoO*_x_* (10 nm) and silver (Ag) (100 nm) were then deposited by evaporation through a shadow mask giving photodiodes with pixel areas of 0.045 cm^2^.

### OTFTs fabrication

Bottom-contact, top-gate OTFTs were fabricated on glass substrates. Substrates were sonicated in Decon 90/deionized water solution for 5 min, followed by sequential sonication in acetone and isopropanol. Gold (40 nm) was deposited via thermal evaporation in high vacuum (10^−6^ mbar) to form the source/drain electrodes, resulting in transistor devices with channel length in the range of 30 to 100 μm and width of 1 mm. For the hole-only TFTs, a self-assembled monolayer, pentafluorothiοphenol (PFBT) was used to treat the workfunction. Substrates with source/drain contacts were immersed in 7 mmol of PFBT solution in isopropanol. No further treatment was conducted for the electron-only TFTs. Organic semiconductors were spin-coated from a solution (5 mg/ml) in anhydrous chlorobenzene at 2000 rpm for 30 s, followed by thermal annealing at 100°C for 10 min. CYTOP (90 nm) was used as dielectric layer, followed by 50-nm thermal evaporated aluminum, which formed the gate electrode. Device fabrication and electrical measurements were performed in a nitrogen glovebox. Transistor characterization was carried out using a Keithley 4200 semiconductor parameter analyzer.

### *J*-*V* measurements

Current density-voltage (*J*-*V*) characteristics were measured using a Keithley 4200 Source-Measure unit (scan rate of 25 mV s^−1^). An Oriel Instruments Solar Simulator with a Xenon lamp and calibrated to a silicon reference cell was used to provide AM1.5G irradiance. For determination of the LDR, a neutral white light LED driven by a function generator (Thorlabs, DC2200) was used. The LED light was attenuated using a selection of neutral density filters placed between the lamp and OPD. The photocurrent (*J*_ph_) was calculated as the difference in response between the illuminated current density (*J*_L_) and dark current density (*J*_d_) at each light intensity. All the devices were tested in nitrogen atmosphere.

### NEP measurements

To calculate the NEP, OPD devices were connected to a lock-in amplifier (MFLI, Zurich Instruments AG) and illuminated with a 940-nm IR light. A frequency of 77 Hz was used and controlled with an optical chopper.

### Responsivity

Responsivity was measured using an integrated system from Quantum Design PV300. All the devices were tested in ambient air.

### Dynamic measurements

Dynamic measurements were performed using a digital oscilloscope (Tektronix, TDS3032B). The PPDs were illuminated with a neutral white light LED driven by a function generator (Thorlabs, DC2200). For determination of the rise and fall time, a 1-kHz square wave pulse was applied to the LED using the function generator. For determination of the cut-off frequency, sinusoidal functions with varying frequencies between 100 Hz and 1 MHz were used to drive the LED. All the devices were tested in nitrogen atmosphere.

### GIWAXS measurements

GIWAXS measurements were performed at the noncrystalline diffraction beamline (BL11-NCD-Sweet) at ALBA Synchrotron Radiation Facility in Barcelona (Spain). A detector (Rayonix, WAXS LX255-HS) with a resolution of 1920 × 5760 pixels was used to collect the scattering signals. Sample holder position was calibrated with chromium oxide (Cr_2_O_3_) standard. The incident energy was 12.4 eV, and the sample-to-detector distance was set at 200.93 mm. The angle of incidence αi was set between 0.1 and 0.15, and the exposure time was 5 s. 2D GIWAXS patterns were corrected as a function of the components of the scattering vector with a MATLAB script developed by A. Nogales and E. Gutiérrez. Thin films were cast onto highly doped silicon substrates following same processing route used for the device fabrication.

### Transient absorption spectroscopy

A broadband femtosecond transient absorption spectrometer Helios (Spectra Physics, Newport Corp.) was used for pump-probe measurements on the neat polymer and acceptor films and their blends. A 1-kHz Ti:Sapphire regenerative amplifier (Solstice, Spectra Physics, Newport Corp.) delivered ultrafast laser pulses (800 nm, <100 fs full width at half maximum) to an optical parametric amplifier (TOPAS Prime, Spectra Physics) and a frequency mixer (Niruvis, Light Conversion) to generate pump pulses at 850 and 1500 nm, which were modulated at 500 Hz by an optical chopper system (Thorlabs). Seed pulses (800 nm) were also delayed on the 6-ns mechanical delay stage and passed through a sapphire crystal to produce a white light probe (400 to 900 nm). Spatial and temporal overlap of the pump and the focused probe beams was achieved on the thin-film samples, contained in a quartz cuvette under a constant flow of nitrogen. The fluences were calculated on the basis of the probe beam size of 0.5 mm^2^ at the sample. Background and chirp corrections were applied to the spectra after measurement using the Surface Xplorer software.

### Photoplethysmography

Photoplethysmography measurements (fig. S16) were performed by directly connecting the OPD devices to a Keithley 4200 Source-Measure unit and recording the current as a function of time upon illumination with different LEDs driven by a function generator (Thorlabs, DC2200).
