# Supplementary material for: Enhanced sub-1 eV detection in organic photodetectors through tuning polymer energetics and microstructure
Source: Sci Adv. 2023 Jun 7;9(23):eadh2694. doi: 10.1126/sciadv.adh2694 (PMC10246890; doi:10.1126/sciadv.adh2694)
Supplement: Supplementary file 1 — Supplementary Text Figs. S1 to S16 Table S1 [file sciadv.adh2694_sm.pdf]

Science Advances
AAAS

Supplementary Materials for

Enhanced sub-1 eV detection in organic photodetectors through tuning polymer energetics and microstructure

Polina Jacoutot *et al.*

Corresponding author: Nicola Gasparini, n.gasparini@imperial.ac.uk

Sci. Adv. **9**, eadh2694 (2023)
DOI: 10.1126/sciadv.adh2694

This PDF file includes:

Supplementary Text
Figs. S1 to S16
Table S1

Supplementary Text

Polymer synthesis

All reactions are air sensitive and, therefore, were performed under argon. All glassware was washed using detergent (Teepol), rinsed with excess water, acetone and methylene dichloride and dried in an oven at 120 °C. All solvents and reagents were purchased from Sigma Aldrich. Toluene was distilled using calcium hydride (CaH_2) and benzophenone prior to polymerization. 4,9-dibromo-6,7-bis(4-((2-decyltetradecyl)oxy)phenyl)-[1,2,5]thiadiazolo[3,4-g]quinoxaline was synthesized according to already published procedures *(55)*. 5,5''-bis(trimethylstannyl)-2,2':5',2''-terthiophene was synthesized based on a modified literature procedure *(56)*.

To a 50 mL 2-neck round bottom flask containing 0.6017 g (0.500 mmol) of 4,9-dibromo-6,7-bis(4-((2-decyltetradecyl)oxy)phenyl)-[1,2,5]thiadiazolo[3,4-g]quinoxaline was added 0.2870 g (0.500 mmol) of 5,5''-bis(trimethylstannyl)-2,2':5',2''-terthiophene, 0.0133 g (0.0145 mmol) of Pd_2dba_3 and 0.0177 g (0.0580 mmol) of tri(o-tolyl)phosphine. The flask was subjected to 3 x vacuum/nitrogen backfill cycles. Then 20 mL of dried toluene was added and the flask was placed in a pre-heated oil bath at 120 °C and stirred for 24 h. Then the reaction was precipitated into 200 mL of methanol and a green solid was collected by filtration. The polymer was then placed in a thimble and subjected to Soxhlet extraction with methanol, acetone, hexane, $CHCl_3$ and chlorobenzene. Then the chlorobenzene solution was concentrated, precipitated into 200 mL of methanol and dark green solid with a metallic shine (0.5080 g, 96 %) was collected by filtration. GPC: M_n = 30800 Da, M_w = 73400 Da, $Đ$ = 2.4.

Gel Permeation Chromatography (GPC)

Average molecular weights per number ($\overline{M_n}$) and polydispersity indices ($Đ$) were determined with GPC at 150 °C on a high temperature PL-GPC 220 system using a PL-GEL 10 μm guard column, two PL-GEL 10μm Mixed-B columns and ortho-dichlorobenzene (*o*-DCB) as the eluent. The instrument was calibrated with narrow polystyrene standards with M_p ranging from 4830 g/mol to 3242000 g/mol.

Air Photoemission Spectroscopy (APS) and Kelvin Probe (KP) measurements on thin films

The workfunction and HOMO of the organic semiconductors was measured using the APS and KP from KP Technology. TQ-T and TQ-3T were spin coated on ITO glass substrates. The HOMO level was extracted from the slope of the cube root of the photoemission signal, as showed in Fig. S2.

Material characterizations

4,9-dibromo-6,7-bis(4-((2-decyltetradecyl)oxy)phenyl)-[1,2,5]thiadiazolo[3,4-g]quinoxaline

^{1}H-NMR (600 MHz, $CDCl_3$, 298 K): δ_H 7.81-7.71 (4H, d, *J*= 7 Hz), 6.97-6.88 (4H, d, *J*=7 Hz) 3.79-3.86 (4H, m), 1.85-1.81 (2H, m), 1.52-1.25 (84H, m), 0.91-0.86 (12H, m).

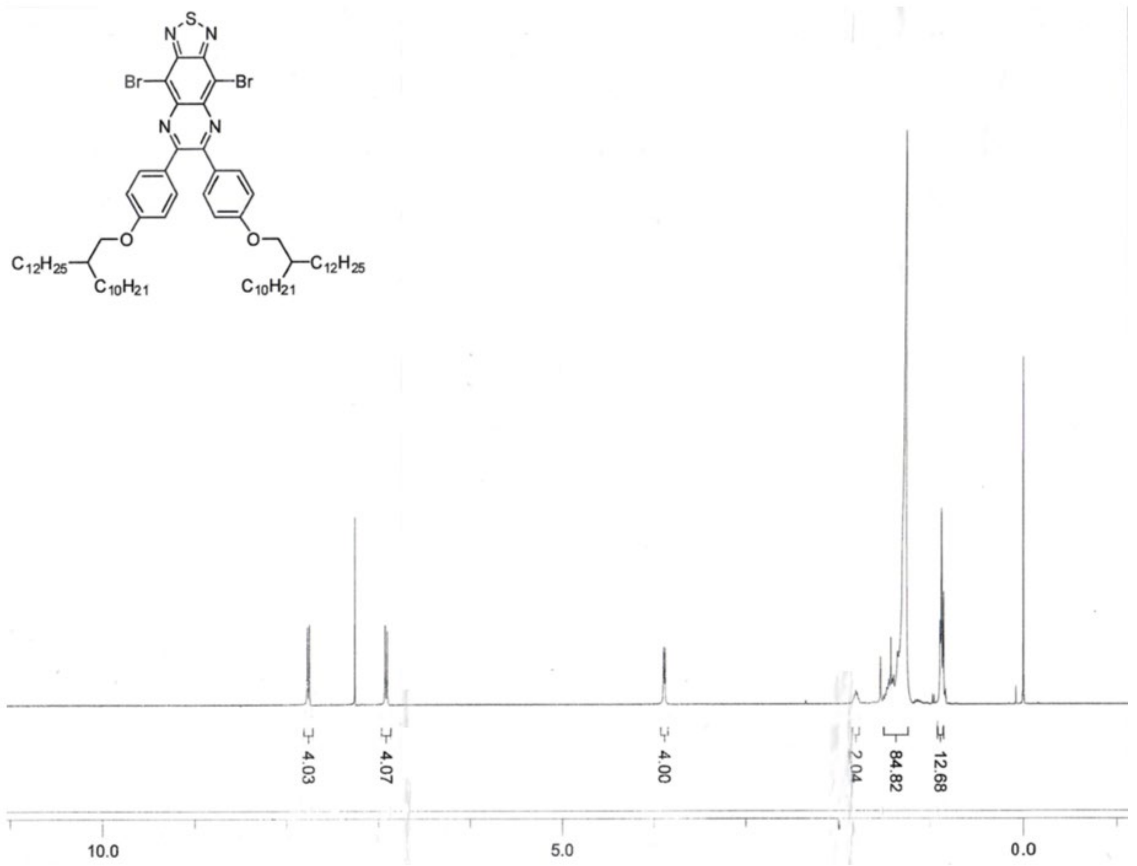

Fig. S1. **Monomer ^{1}H-NMR.** ^{1}H-NMR spectrum of 4,9-dibromo-6,7-bis(4-((2-decyltetradecyl)oxy)phenyl)-[1,2,5]thiadiazolo[3,4-g]quinoxaline.

5,5''-bis(trimethylstannyl)-2,2':5',2''-terthiophene

^{1}H-NMR (600 MHz, $CDCl_3$, 298 K): δ_H 7.47-7.45 (4H, m), 7.28 (2H, d, *J*= 5.0 Hz).

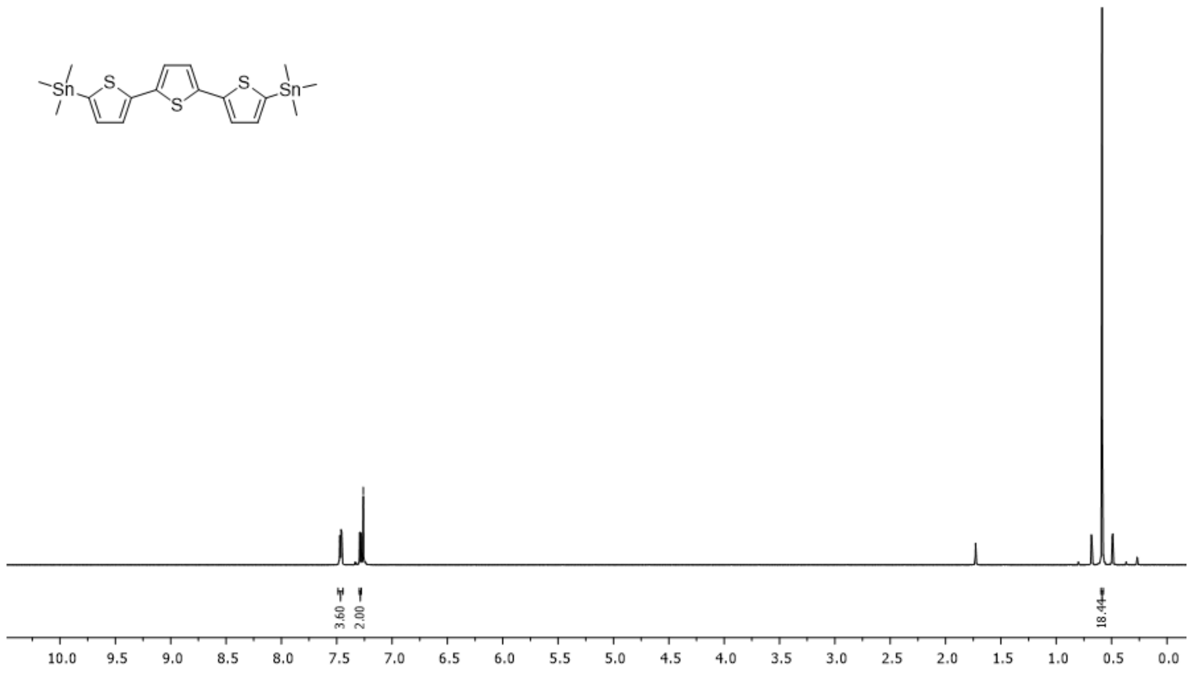

Fig. S2. **Monomer 2 ^{1}H-NMR.** ^{1}H-NMR spectrum of 5,5''-bis(trimethylstannyl)-2,2':5',2''-terthiophene

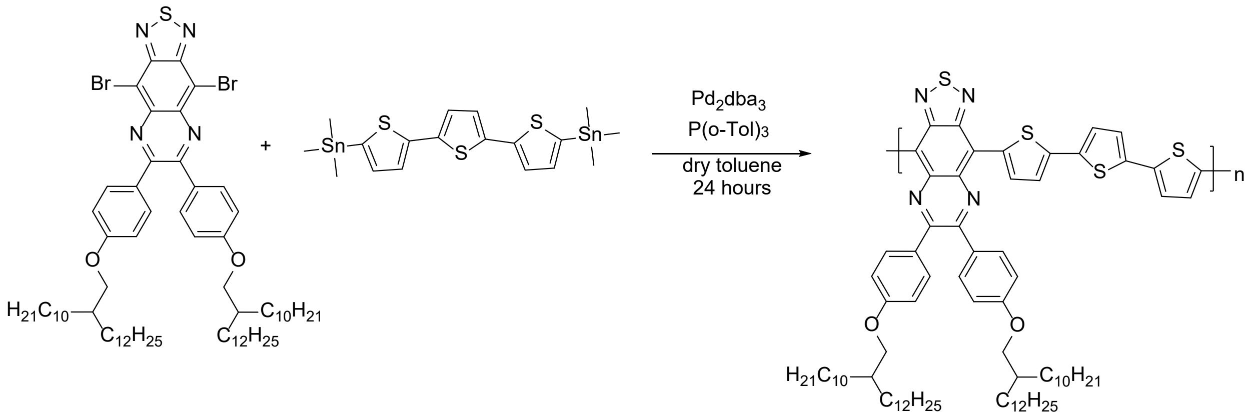

Fig. S3. **Synthesis of TQ-3T.** Synthetic route for TQ-3T.

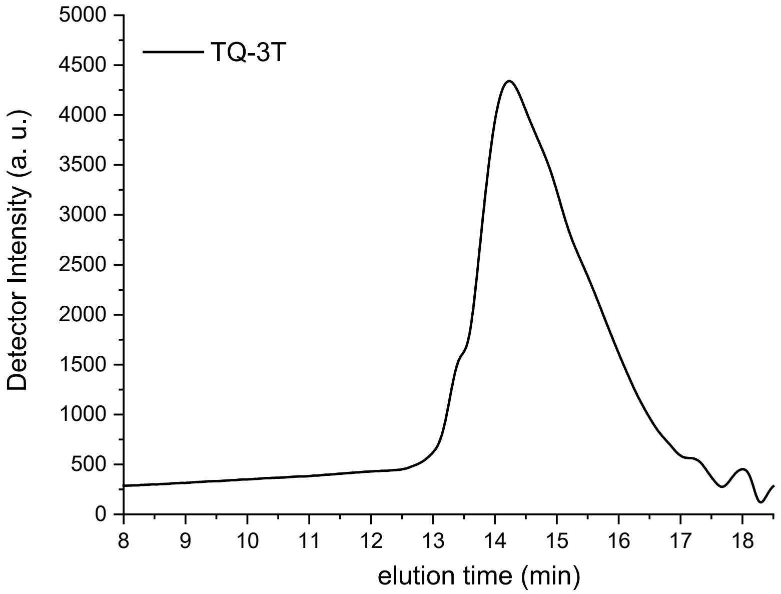

Fig. S4. **GPC measurement.** Gel permeation chromatograph of the TQ-3T obtained using ortho-dichlorobenzene (oDCB) as eluent at flow rate of 1 mL/min and at 150 ºC

^{1}H-NMR (600 MHz, $CDCl_3$, 298 K): δ_H 7.92-7.36 (6H, m), 7.13-6.50 (8H, m), 4.20-3.82 (4H, m), 2.36-1.04 (86H, m), 1.52-1.25 (84H, m), 0.98-0.70 (12H, m).

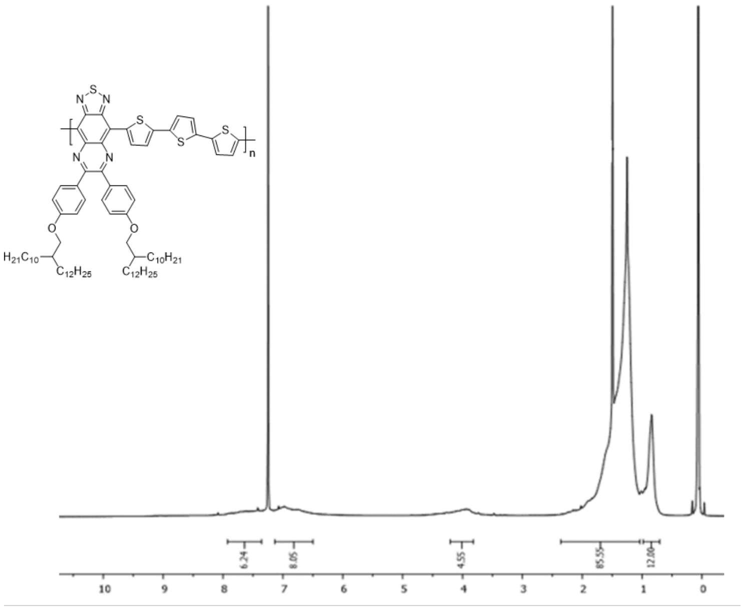

Fig. S5. **^{1}H-NMR TQ-3T.** ^{1}H-NMR spectrum of TQ-3T

^{13}C-NMR (150 MHz, $CDCl_3$, 298 K): 159.8, 150.8, 134.2, 132.0, 131.0, 113.0, 112.5, 70.4, 37.3, 31.0, 30.4, 39.2, 28.8, 28.5, 26.5, 26.0, 21.7, 13.1.

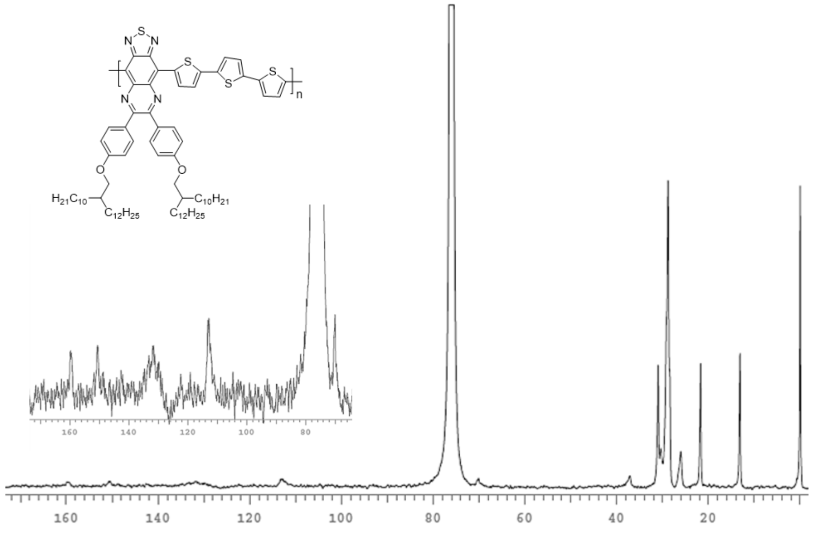

Fig. S6. **^{13}C-NMR TQ-3T.** ^{13}C-NMR spectrum of TQ-3T

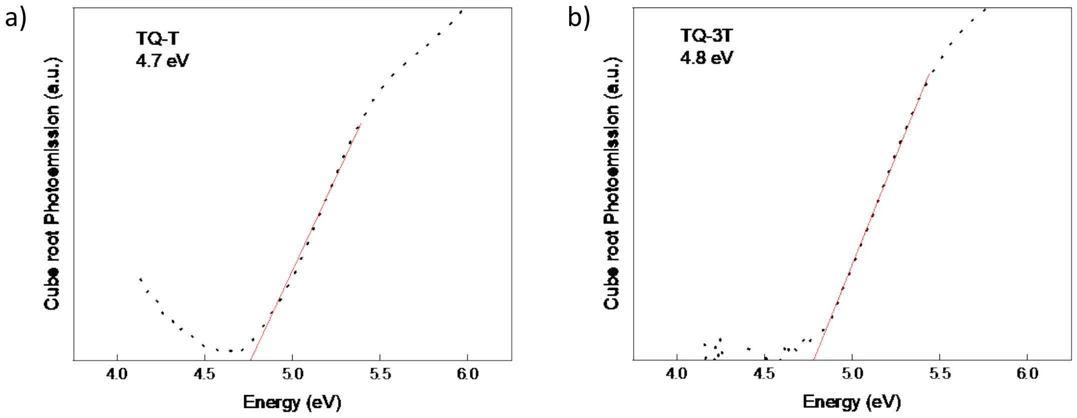

Fig. S7. PESA measurements. Cube root of the photoemission signals for (a) TQ-T and (b) TQ-3T thin films.

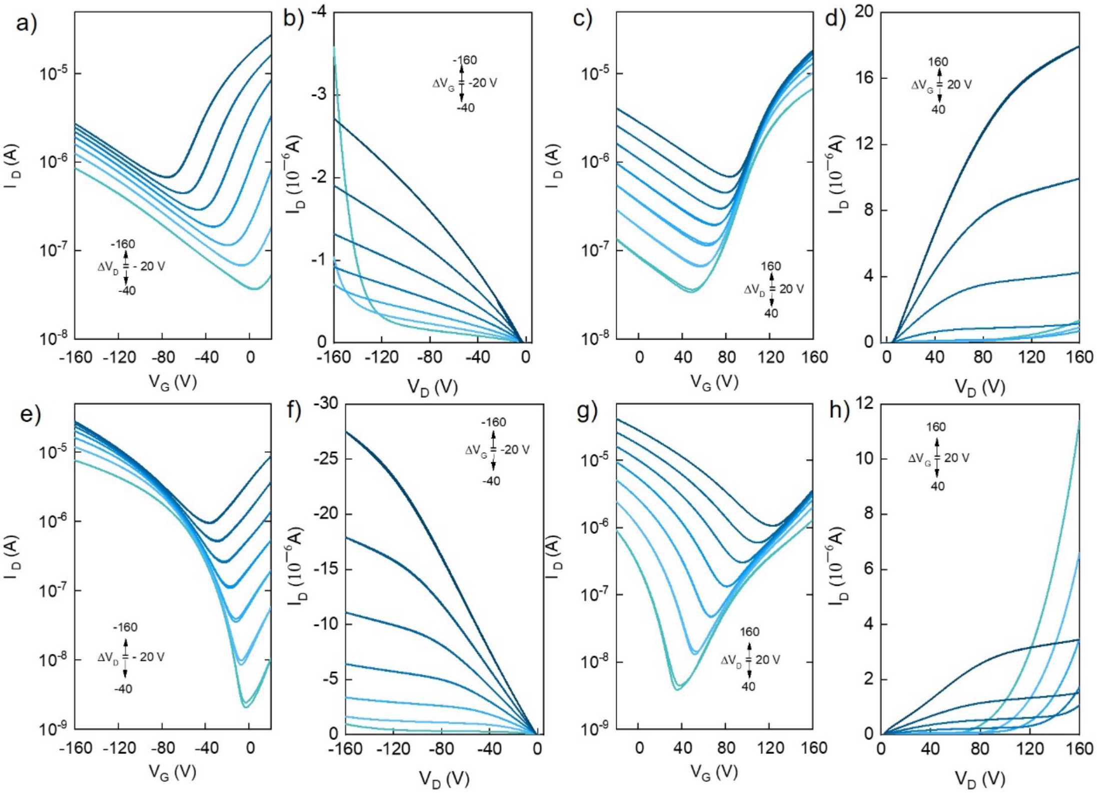

Fig. S8. OTFT measurements. Representative transfer (a-c) and output characteristics (b-d) of TQ-T showing hole and electron transport, respectively. Representative transfer (e-g) and output characteristics (f-h) of TQ-3T showing hole and electron transport, respectively.

Table S1. Mobility values. Carrier mobility as extracted from low bandgap polymers measured from BC-TG OTFTs in the saturation regime (V_D=±160V).

OTFT	carrier type	saturation mobility cm^2/Vs
TQ-T	electron	$3x10^{-2}$
	hole	$3.5x10^{-3}$
TQ-3T	electron	$3x10^{-3}$
	hole	$7x10^{-2}$

OPD Measurements

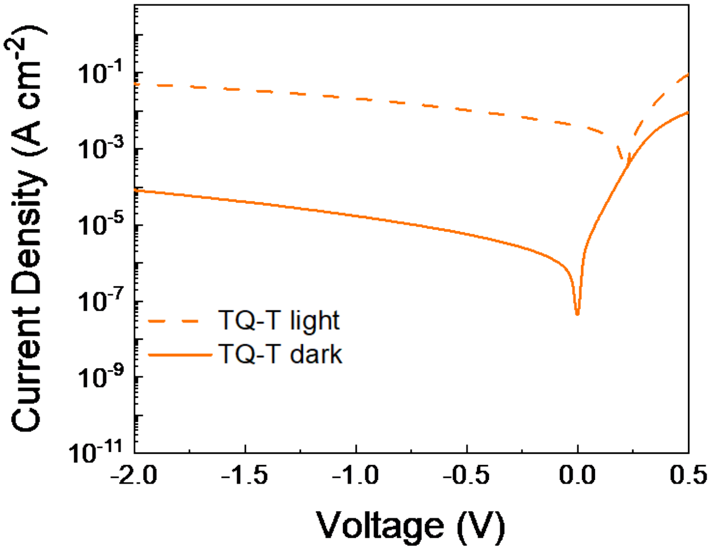

Fig. S9. J-V characteristics of TQ-T:IEICO-4F. JV curves for TQ-T (orange)- based OPDs under AM 1.5G illumination and in the dark.

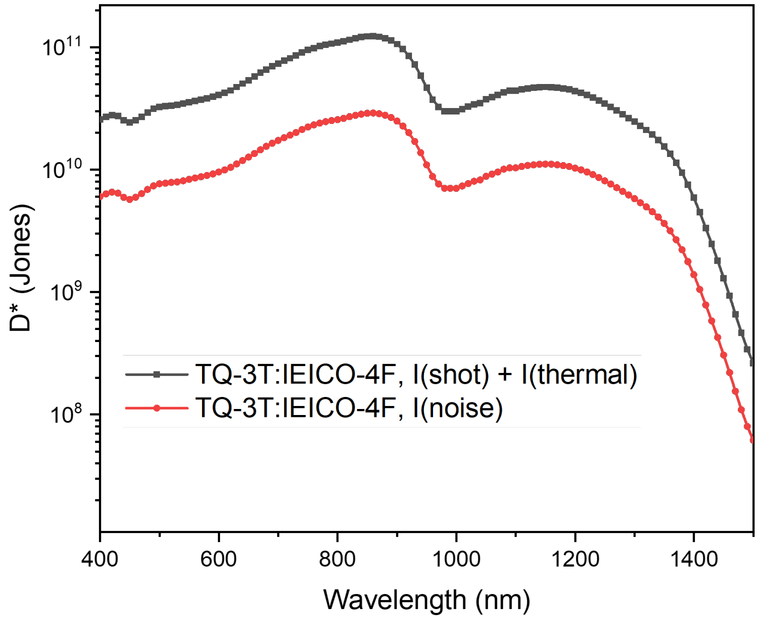

Fig. S10. Specific detectivity plots. Specific detectivity of TQ-3T:IEICO-4F calculated from the shot and thermal noise (black) and calculated from the noise measured with the oscilloscope (red).

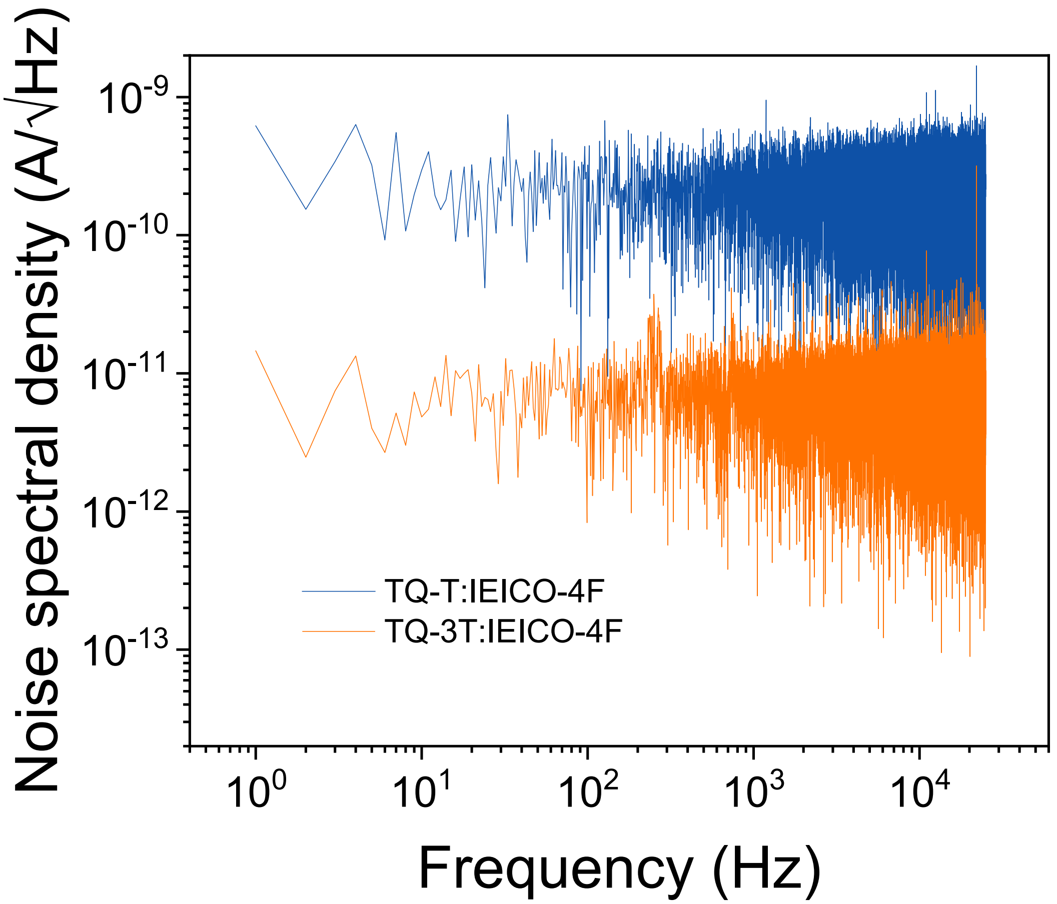

Fig. S11. NEP measurements. Noise spectral density of TQ-3T and TQ-T-based devices.

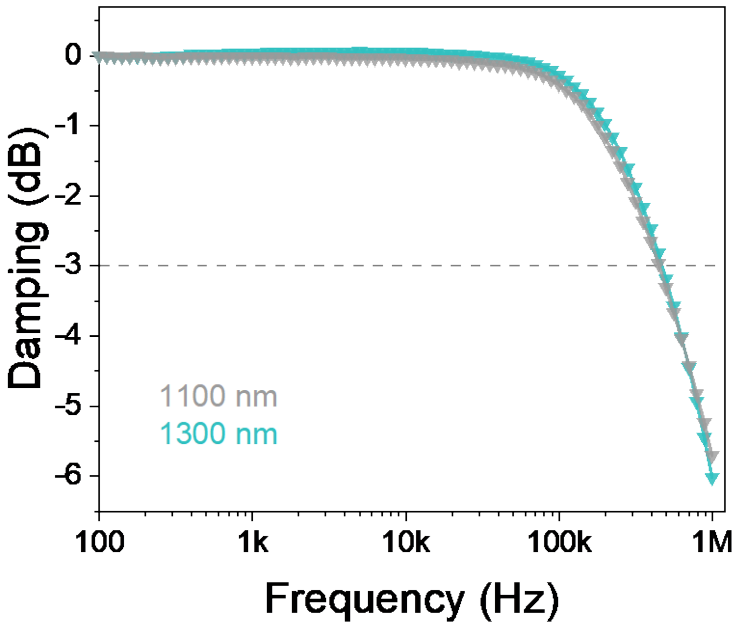

Fig. S12. Cut-off frequency measurements. Cut-off frequency of TQ-3T:IEICO-4F under 1100 nm and 1300 illumination and -2 V applied bias.

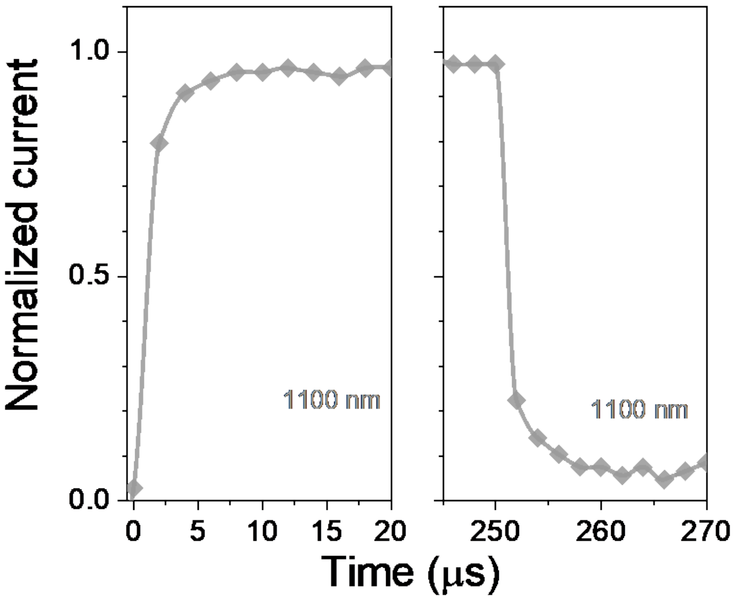

Fig. S13. Transient measurements. Raise and fall time of TQ-3T:IEICO-4F under 1100 nm and 1300 illumination and -2 V applied bias.

Transient Absorption Spectroscopy

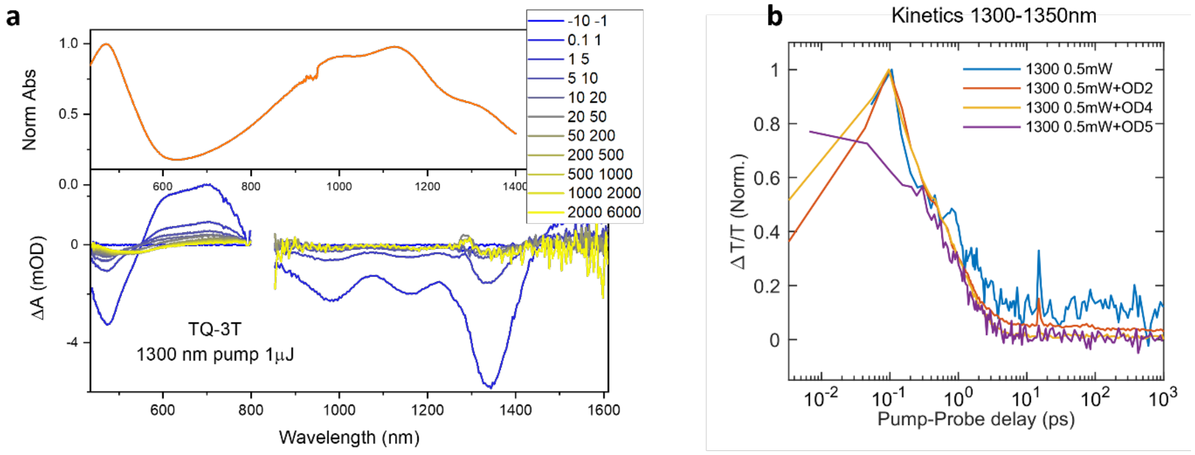

Fig. S14. Transient absorption spectroscopy. a) Steady-state (top) and transient (bottom) absorption spectra of the neat TQ-3T polymer with the (b) associated normalized exciton kinetics at 1300-1350 nm under different pump powers.

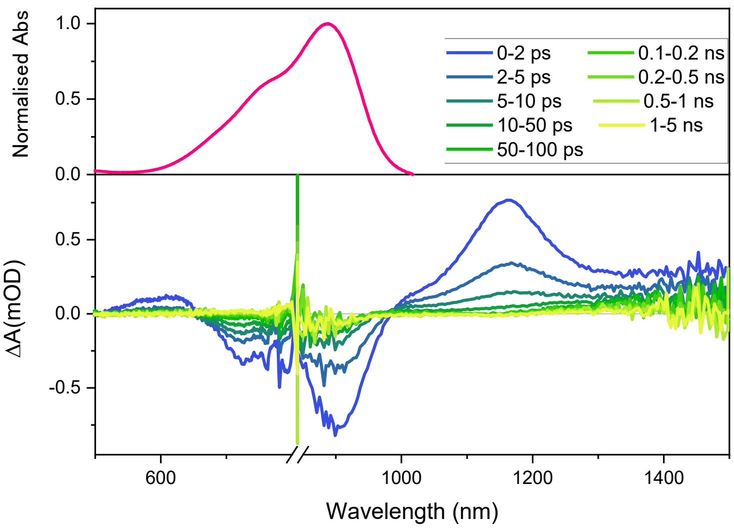

Fig. S15. TAS of IEICO-4F. Steady-state (top) and transient (bottom) absorption spectra of the neat IEICO-4F under low intensity (1 μJ) illumination with 800 nm pump light.

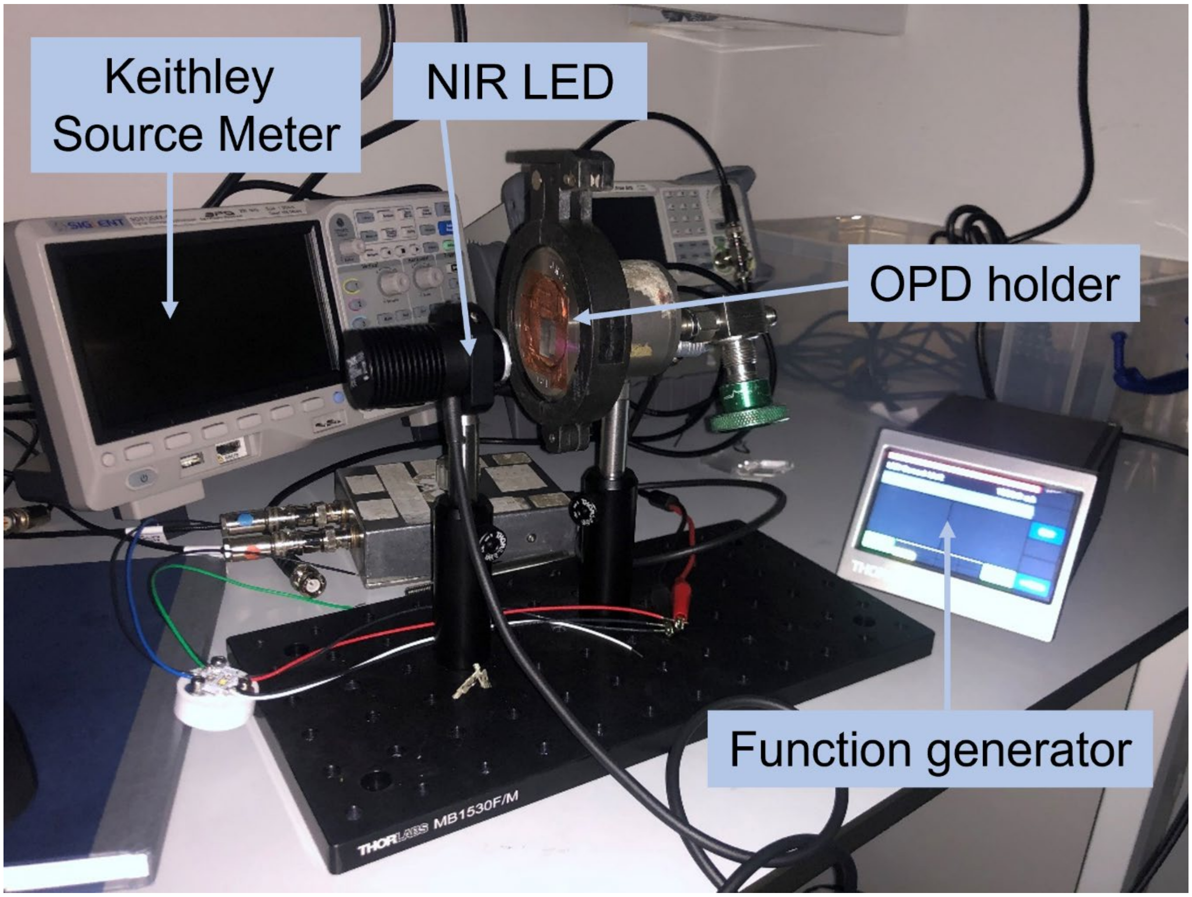

Fig. S16. Home-made PPG setup. The PPG setup for real-time current read-out without signal amplification.
